# Неонатальный скрининг врожденной дисфункции коры надпочечников в России: итоги десятилетия (2015–2025) и уроки клинических случаев

**DOI:** 10.14341/probl13758

**Published:** 2026-05-20

**Authors:** М. В. Воронцова, И. С. Чугунов, И. В. Копылова, А. Э. Шаповалова, А. М. Горбачева, В. А. Петеркова, Н. Г. Мокрышева

**Affiliations:** Московский государственный университет имени М.В. Ломоносова; Lomonosov Moscow State University; Национальный медицинский исследовательский центр эндокринологии им. академика И.И. Дедова; Endocrinology Research Centre

**Keywords:** врожденная дисфункция коры надпочечников, неонатальный скрининг, дефицит 21-гидроксилазы, congenital adrenal hyperplasia, neonatal screening, 21-hydroxylase deficiency

## Abstract

Неонатальный скрининг на врожденную дисфункцию коры надпочечников (ВДКН) проводится в России с 2006 г. За 2015–2025 гг. скрининг охватил 15 546 274 новорожденных и позволил выявить 1871 ребенка с классическими формами дефицита 21-гидроксилазы. Средняя частота выявляемости составила 1:8309 (0,012% или 1,20 на 10 000 в год) с ежегодными колебаниями от 1,03 до 1,48 на 10 000 новорожденных в год. Установлена выраженная межрегиональная вариабельность заболеваемости: максимальные значения зафиксированы в Карачаево-Черкесской Республике (6,16/10 000 в год), Ленинградской области (4,04/10 000 в год) и Приморском крае (3,07/10 000 в год), среди федеральных округов лидирует Уральский (0,017% от всех скринированных новорожденных). Сроки диагностики обуславливают критические различия в исходах, включая риск развития кризов надпочечниковой недостаточности. Неонатальный скрининг подтверждает свою высокую эффективность, а выявленные региональные различия обосновывают необходимость дальнейших популяционно-генетических исследований для оптимизации медико-генетического консультирования.

## Abstract (en)

Neonatal screening for congenital adrenal hyperplasia (CAH) has been conducted in Russia since 2006. An analysis of results from 2015–2025, covering 15,546,274 newborns, identified 1,871 children with classic forms of 21-hydroxylase deficiency. The average detection rate was 1:8,309 (0.012% or 1,20 per 10 000 per year) with annual variations ranging from 1.03 to 1.48 per 10,000 newborns per year. Significant interregional variability in incidence was observed, with the highest rates recorded in the Karachay-Cherkess Republic (6.16/10,000 per year), Leningrad Oblast (4.04/10,000 per year), and Primorsky Krai (3.07/10,000 per year). Among federal districts, the Urals Federal District ranked highest. The timing of diagnosis leads to critical differences in outcomes, including the risk for adrenal crisis. Newborn screening confirms its high efficiency, and the identified regional variations underscore the need for further population genetic studies to optimize medical genetic counseling.

## ВВЕДЕНИЕ

Неонатальный скрининг является важным инструментом ранней диагностики врожденных заболеваний, позволяющим выявить патологические нарушения на доклинической стадии, своевременно начать терапию и предотвратить жизнеугрожающие состояния. История неонатального скрининга началась в 1960-х годах с внедрения Робертом Гатри теста для раннего выявления фенилкетонурии. Ключевой инновацией стало использование образцов капиллярной крови, высушенных на фильтровальной бумаге, что позволило стандартизировать и централизовать массовое обследование новорожденных [1]. В дальнейшем этот подход стал моделью для разработки программы скрининга других тяжелых наследственных заболеваний, включая врожденную дисфункцию коры надпочечников.

Врожденная дисфункция коры надпочечников (ВДКН) — группа заболеваний с аутосомно-рецессивным типом наследования, обусловленных дефектом ферментов или транспортных белков, участвующих в биосинтезе кортизола в коре надпочечников. Наиболее распространенной формой ВДКН является дефицит 21-гидроксилазы, при нарушении функции которой нарушается синтез как кортизола, так и альдостерона в большинстве случаев. Специфическим маркером данной формы ВДКН служит повышение концентрации 17-гидроксипрогестерона (17-OHP). Клинические проявления варьируют в зависимости от степени недостаточности фермента и включают гипогликемический синдром, вирилизацию наружных гениталий у девочек, симптомы гиперандрогении у мальчиков и девочек, а также развитие сольтеряющего синдрома, проявляющегося гипонатриемией, гиперкалиемией, дегидратацией и артериальной гипотензией [2][3][4]. При отсутствии своевременно начатой заместительной терапии глюко- и минералокортикоидами у пациентов с ВДКН резко возрастает риск развития криза надпочечниковой недостаточности, потенциально приводящего к летальному исходу в раннем неонатальном периоде.

Впервые скрининг на ВДКН был реализован в 1977 г. в штате Аляска, где наблюдалась высокая распространенность данной патологии среди коренного населения — эскимосов. В рамках экспериментальной программы методом радиоиммунного анализа был исследован уровень 17-гидроксипрогестерона (17-OHP) в сухих пятнах капиллярной крови на фильтровальной бумаге, взятых из пятки у 53 357 новорожденных. Результаты пилотного проекта продемонстрировали возможность проведения и эффективность неонатального скрининга ВДКН [3][5][6].

Основанием для внесения заболевания в программу расширенного неонатального скрининга служит соответствие его ряду установленных требований: потенциальная угроза жизни в условиях отсутствия терапии, достаточно высокая распространенность среди населения страны, наличие чувствительного и специфичного диагностического теста, наличие эффективного лечения, а также готовность национальной системы здравоохранения обеспечить последующее ведение пациента. ВДКН полностью удовлетворяет этим требованиям, так как является относительно распространенным орфанным заболеванием, с потенциально летальным исходом в первые недели или месяцы жизни ребенка, для диагностики которого достаточно исследования одного специфического маркера финансово не затратной тест-системой. Это обусловило включение ВДКН в национальные программы скрининга в ряде стран [7][8]. Согласно данным, полученным при анализе международных программ неонатального скрининга за 1980–1988 годы, частота выявления классической формы ВДКН составила 1 случай на 14 199 живорожденных. При этом сольтеряющая форма регистрировалась приблизительно в три раза чаще, чем простая вирильная. Кроме того, была зафиксирована более высокая общая заболеваемость по сравнению с предыдущими эпидемиологическими оценками, что свидетельствовало о повышении выявляемости случаев ВДКН в результате введения неонатального скрининга [8]. На сегодняшний день скрининг новорожденных на ВДКН в рамках национальных программ или пилотных проектов внедрен более чем в 40 странах мира [9][10]. Тотальный неонатальный скрининг на ВДКН позволяет вовремя диагностировать тяжелые сольтеряющие формы заболевания и исключить ошибки в определении половой принадлежности у девочек.

В Российской Федерации внедрение неонатального скрининга проходило поэтапно: в 1972 г. была создана первая отечественная программа для выявления фенилкетонурии, затем, с 1993 г., два заболевания — фенилкетонурия и врожденный гипотиреоз — был включены в национальную скрининговую программу В 2006 г. в рамках национального проекта «Здоровье» в перечень патологий были включены галактоземия, муковисцидоз и ВДКН (дефицит 21-гидроксилазы). Организация и реализация программы скрининга в России на протяжении последних 20 лет основывалась на нормативных положениях приказа Минздравсоцразвития России от 22.03.2006 № 185 «О массовом обследовании новорожденных детей на наследственные заболевания», устанавливающего единый протокол обследования [11]. Согласно указанным стандартам, забор капиллярной крови из пятки доношенного новорожденного осуществлялся на 4‑е сутки жизни, у недоношенных — на 7–14‑е сутки; для иммунологического исследования 17-OHP рекомендовано использовать строго регламентированные коммерческие наборы с целью формирования централизованной системы мониторинга.

В связи с расширением перечня заболеваний, включенных в неонатальный скрининг, а также тенденцией к сокращению срока пребывания детей в родильном доме, приказом Министерства здравоохранения России от 21.04.2022 №274н «Об утверждении Порядка оказания медицинской помощи пациентам с врожденными и (или) наследственными заболеваниями» [12] пересмотрены сроки проведения исследования: рекомендовано производить забор крови в течение первых 24–48 часов жизни у доношенных и на 7‑е сутки (144–68 часов) у недоношенных.

Несмотря на логистические преимущества и возможность минимизировать пропуски случаев заболеваний, смещение сроков забора биоматериала на более ранний постнатальный период сопряжено с увеличением частоты ложноположительных результатов в отношении ВДКН. Это обусловлено физиологическим подъемом концентрации стероидных гормонов, в том числе 17-OHP, в первые сутки жизни, особенно у недоношенных детей и детей с тяжелой соматической патологией [13]. Помимо этого, причиной ложноположительных результатов может быть относительно низкая специфичность антител и их перекрестная реакция с метаболитами стероидогенеза в ходе иммуноферментного анализа [6]. С целью повышения специфичности диагностических тестов в 2000-х годах началось активное внедрение второго этапа неонатального скрининга на ВДКН методом тандемной масс-спектрометрии (ТМС, или LC-MS/MS). Первый опыт применения ТМС продемонстрировал снижение количества ложноположительных результатов, особенно в группе недоношенных новорожденных [14]. В последующие годы метод внедрен в национальные программы неонатального скрининга в ряде стран. Современные клинические рекомендации, в том числе отечественные, предполагают использование ТМС в качестве второго этапа при выявлении положительного результата скрининга или пограничных значений 17-OHP. Помимо этого, ТМС позволяет диагностировать и редкие формы ВДКН [15].

Помимо программы скрининга, золотым стандартом для окончательной постановки диагноза является молекулярно-генетическое тестирование, где оно технически доступно. Очевидно, что только генетические методы могут определить точную мутацию в исследуемом гене даже в случаях вирильной и неклассической форм. Вместе с тем, как молекулярно-генетический анализ, так и мультистероидная ТМС, имеют ограниченную доступность, что осложняет их широкое использование.

Реализация и мониторинг неонатального скрининга в Российской Федерации требуют координации между государственными структурами, лабораториями и клиническими подразделениями с целью доступности тестирования для всех новорожденных, своевременного информирования семей и назначения соответствующей терапии. На текущий момент на основании приказа Министерства здравоохранения России от 21.04.2022 № 274н «Об утверждении Порядка оказания медицинской помощи пациентам с врожденными и (или) наследственными заболеваниями» [12] в субъектах Российской Федерации приняты территориальные ведомственные акты, регламентирующие проведение неонатального и расширенного неонатального скрининга. Данные нормативные документы адаптируют федеральные требования к региональным условиям и обеспечивают организацию скрининга — порядок проведения, маршрутизацию пациентов, взаимодействие между медицинскими организациями и использование информационных систем.

## НЕОНАТАЛЬНЫЙ СКРИНИНГ ВДКН В РОССИЙСКОЙ ФЕДЕРАЦИИ В 2015–2025 ГГ.

Всего за указанный период скринированы на ВДКН 15 546 274 новорожденных, выявлен 1871 ребенок с данной патологией (0,00012%; 1:8 309 или 1,20 на 10 000). «Тепловая карта» распределения числа детей с ВДКН по субъектам Российской Федерации за период с 2015 по 2025 гг. представлена на рисунке 1.

**Figure:**
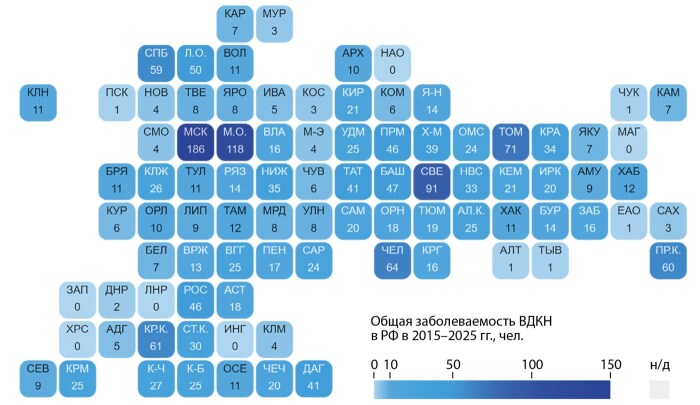
Рисунок 1. Общая заболеваемость ВДКН в РФ в 2015–2025 гг., чел.

Ежегодная заболеваемость колебалась в пределах 1,03–1,48 случая на 10 тыс. новорожденных в год. Динамика заболеваемости представлена на рисунке 2.

**Figure:**
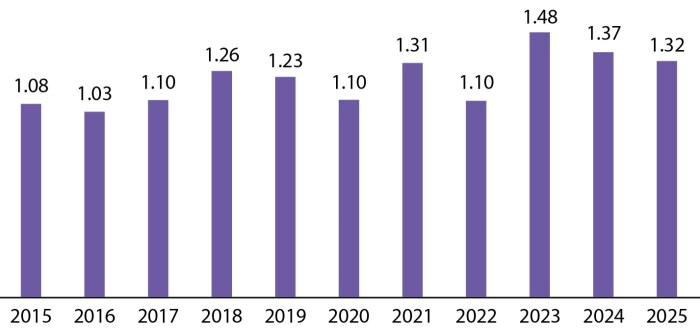
Рисунок 2. Заболеваемость ВДКН в России в 2015–2025 гг. на 10 тыс. новорожденных в год.

Обращает на себя внимание отличие в абсолютной заболеваемости между различными субъектами России. При анализе относительной заболеваемости (доли выявленных случаев заболевания относительно всех скринированных новорожденных) отличия также выявляются, однако меняются субъекты-лидеры (рис. 3).

**Figure:**
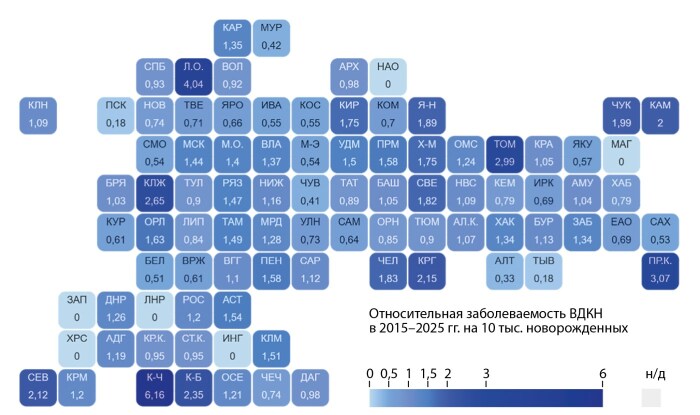
Рисунок 3. Относительная заболеваемость ВДКН в 2015–2025 гг. в разрезе субъектов РФ, на 10 тыс. новорожденных в год.

Исследование, посвященное анализу выявляемости ВДКН в ходе неонатального скрининга в 2010–2015 гг., ранее было проведено Каревой М.А. и соавт. За тот период времени скрининг охватил более 9 млн новорожденных. Частота выявления классических форм 21-гидроксилазной недостаточности в РФ составила 1:9638, что превышало среднемировую распространенность заболевания (1:14 198). Кроме того, были установлены значительные межрегиональные различия в частоте выявления ВДКН в России (от 1:14 876 до 1:6749) [16]. К сожалению, в указанной работе детализированная информация была приведена только по федеральным округам.

При анализе данных за 2015–2025 гг. по федеральным округам наибольшее число случаев ВДКН было зарегистрировано в ЦФО (рис. 4).

**Figure:**
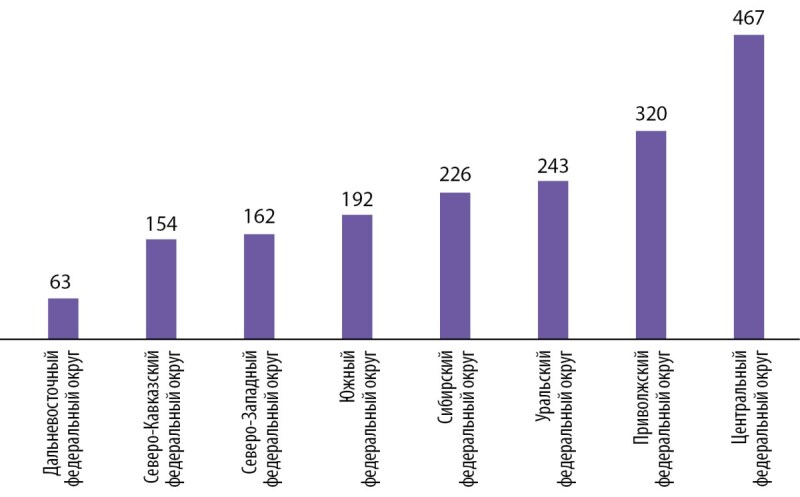
Рисунок 4. Абсолютная заболеваемость ВДКН в России в 2015–2025 гг. по данным неонатального скрининга в разрезе федеральных округов, чел в год.

При этом в относительном выражении наибольшая заболеваемость отмечена в Уральском федеральном округе (рис. 5). Это в целом согласуется с данными Каревой М.А. [16].

**Figure:**
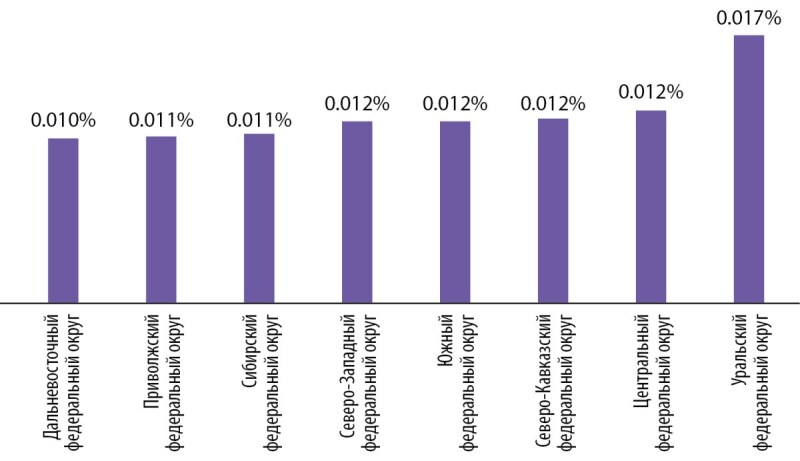
Рисунок 5. Относительная заболеваемость ВДКН в России в 2015–2025 гг. по данным неонатального скрининга в разрезе федеральных округов, % от скринированных новорожденных.

В разрезе субъектов в интервале 2015–2025 гг. наибольшая заболеваемость (6,16 случая на 10 тыс. новорожденных в год) определялась в Карачаево-Черкесской Республике; также высокая заболеваемость наблюдается в Ленинградской области (4,04 случая на 10 тыс. новорожденных в год), Приморском крае (3,07/10 тыс. в год). Полученные данные можно сопоставить со сведениями о национальном составе субъектов РФ, полученными Росстат в ходе Всероссийской переписи населения 2020 г. [17].

В Карачаево-Черкесской Республике на 2020 г. проживало 469 865 человек, из них на вопрос о национальной принадлежности ответили 463 268 (98,6%). Из их числа 44% респондентов считали себя карачаевцами, 28% — русскими, 13% — черкесами, 4% — ногайцами. Остальных национальностей в субъекте было менее 1%.

В Ленинградской области на 2020 г. проживало 2 000 997 человек, из них на вопрос о национальной принадлежности ответили 1 752 846 (87,6%). Из них 94% респондентов относили себя к русским, 1% — к украинцам, остальных национальностей в субъекте было менее 1%.

В Приморском крае на 2020 г. проживало 1 845 165 человек, из них на вопрос о национальной принадлежности ответили 1 473 979 (79,9%). Из них 94% респондентов считали себя русскими, 1% — украинцами, 1% — корейцами. Остальных национальностей в субъекте было менее 1%.

Таким образом, говорить об однозначной связи более высокой заболеваемости с размерами субъекта и/или национальным составом (с тенденцией к созданию семей в более узких социальных группах) на сегодняшний день не представляется возможным. Для определения причин выявленных закономерностей необходим анализ как семей, в которых рождаются дети с ВДКН, так и популяционный анализ носительства рецессивных мутаций, обусловливающих наличие заболевания, в разных этнических группах.

Подобные работы имели бы принципиальное значение для организации медико-генетического консультирования при планировании беременности у пациенток групп высокого риска рождения детей с ВДКН.

## КЛИНИЧЕСКАЯ ЗНАЧИМОСТЬ СВОЕВРЕМЕННОЙ ДИАГНОСТИКИ ВДКН

Диагностика ВДКН с первых дней жизни в рамках неонатального скрининга является крайне важной для своевременного начала лечения и предотвращения сольтеряющего криза. Данное заболевание является потенциально летальным, в связи с чем требует незамедлительного начала заместительной терапии гидрокортизоном и — при наличии минералокортикоидной недостаточности — флудрокортизоном. До появления неонатального скрининга у девочек диагноз чаще устанавливался сразу после рождения на основании вирилизации наружных половых органов, в то время как у мальчиков классические формы ВДКН устанавливались лишь после развития сольтеряющего криза. Эффективность неонатального скрининга не вызывает сомнений [5][8][18][19]. По данным литературы, до введения скрининга неонатальная смертность от сольтеряющего криза ввиду отсутствия правильной верификации диагноза варьировала от 4 до 10%, а также фиксировалось большое число случаев неправильного определения пола у девочек (до 10% случаев) [20]. После введения неонатального скрининга, в том числе в России, соотношение диагностированных случаев ВДКН у мальчиков и девочек сравнялось, и повысилась заболеваемость по сравнению с предыдущими эпидемиологическими данными, что свидетельствует о большей выявляемости данной патологии [5][8][16][18][19].

Неправильное строение наружных половых органов у пациенток женского пола может приводить к ошибочному выбору мужской половой принадлежности, что в дальнейшем создает значительные трудности для родителей в отношении социализации ребенка. У пациентов с вирильной формой заболевания клиническая картина может проявляться не так ярко в первый год жизни, то есть могут отсутствовать сольтеряющие кризы и явные признаки вирилизации половых органов. Вместе с тем сохраняются остальные клинические проявления, такие как гонадотропиннезависимое преждевременное половое развитие, прогрессия вирильного синдрома и гирсутизма у девочек, раннее закрытие зон роста, что в итоге приводит к низкорослости и психосоциальной дезадаптации. Длительное отсутствие заместительной терапии может проводить к повреждению яичек в связи с развитием эктопической ткани надпочечника в яичках (testicular adrenal rest tumors — TART), что в дальнейшем в значительной степени снижает репродуктивный потенциал молодых людей с ВДКН [21–23].

## Клинический пример 1

Пациент С., 6 лет, впервые поступил в ФГБУ «НМИЦ эндокринологии им. академика И.И. Дедова» Минздрава России с жалобами на высокие темпы роста, значительное опережение в росте сверстников. Со слов родителей было известно, что в роддоме были взяты образцы крови в рамках неонатального скрининга, однако результаты родителями получены не были.

С первого года жизни отмечались высокие темпы роста, значительное опережение в росте сверстников, появление лобкового оволосения, однако родители к детскому эндокринологу не обращались.

Диагноз «ВДКН, дефицит 21-гидроксилазы, вирильная форма» установлен только в возрасте 5 лет на основании выраженного ускорения роста (SDS роста +2.8), ускорения полового развития (на момент осмотра половое развитие соответствовало Таннер 2 (G2, P1), значительного ускорения костного возраста (соответствовал 13 годам), повышения концентрации 17-ОНР до 798 нмоль/л (0,2–3,2). Диагноз был подтвержден молекулярно-генетически, выявлены компаунд-гетерозиготные патогенные вариантные замены c.293-13C>G c.518T>A, p.I173N в гене CYP21A2. Была назначена заместительная терапия гидрокортизоном в дозе 12,5 мг/м²/сут, на фоне терапии концентрации 17-ОНР нормализовались.

Как осложнение длительной декомпенсации заболевания, снижение концентраций надпочечниковых андрогенов на фоне терапии гидрокортизоном при значительном опережении костного возраста привело к преждевременной активации гипоталамо-гипофизарно-гонадной оси и развитию преждевременного гонадотропинзависимого полового развития, которое было диагностировано в возрасте 6 лет на основании увеличения размеров тестикул до 6 мл и данных стимуляционной пробы с аналогом гонадотропин-рилизинг-гормона (ГнРГ), где максимальный выброс ЛГ составил 12 Ед/л (норма — менее 6 Ед/л). По данным УЗИ органов мошонки были выявлены множественные парацентральные образования в тестикулах размером до 1,5 см, неправильной формы, гипоэхогенные, с внутриузловым кровотоком, которые были расценены как вторичные образования яичек, представляющие собой гиперплазию эктопированной надпочечниковой ткани (TARTs). При проведении МРТ органов мошонки — признаки очаговых образований обоих яичек, соответствующие TART (рис. 6).

**Figure:**
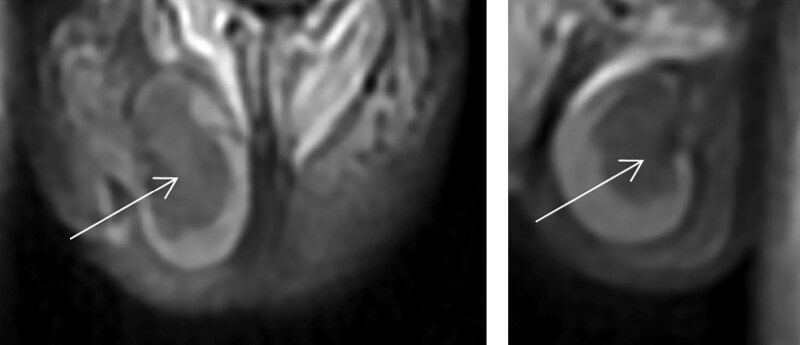
Рисунок 6. Двусторонние TARTs у пациента С. с ВДКН.

Учитывая прогрессирующее гонадотропинзависимое половое развитие и, как следствие, значительное ухудшение ростового прогноза (конечный прогнозируемый рост с учетом костного возраста составлял 156 см при целевом среднеродительском росте 182,5 см), была инициирована терапия аналогами ГнРГ пролонгированного действия в дозе 3,75 мг 1 раз в 28 дней.

Таким образом, поздняя диагностика ВДКН вне процедуры неонатального скрининга привела к снижению конечного прогнозируемого роста, формированию психосоциальной дезадаптации ребенка вследствие преждевременного полового развития и потребовала назначения дополнительных препаратов в попытке достичь социально приемлемого роста для пациента. Длительная декомпенсация заболевания вызвала образование TART в тестикулах, что в дальнейшем может привести к снижению репродуктивного потенциала у данного пациента.

В случае своевременно поставленного диагноза, адекватно подобранной заместительной терапии надпочечниковой недостаточности глюко- и минералокортикоидами и удовлетворительного контроля концентраций надпочечниковых андрогенов с достижением их супрессии физическое развитие и возраст начала полового созревания пациентов с ВДКН обычно соответствуют данным показателям в общей популяции [2–4][16][21][24].

## Клинический пример 2

Пациентка Д., 17 лет, впервые была обследована в ФГБУ «НМИЦ эндокринологии им. академика И.И. Дедова» Минздрава России в возрасте 5 месяцев.

На основании неправильного строения наружных гениталий (вирилизация 3 степени по шкале Прадера) в роддоме ребенок был зарегистрирован в мужском поле. С рождения отмечались частые срыгивания, отсутствие прибавки в весе, а со второй недели жизни — рвота фонтаном. После получения результатов неонатального скрининга (17ОНР — 458,2 нмоль/л (10–35) был установлен диагноз «ВДКН, дефицит 21-гидроксилазы, сольтеряющая форма», инициирована терапия гидрокортизоном и флудрокортизоном. По данным цитогенетического исследования был определен нормальный женский кариотип 46ХХ. Учитывая клинический диагноз, паспортный пол был изменен на женский.

Диагноз был подтвержден в возрасте 1 месяца жизни по результатам молекулярно-генетического исследования: выявлена патогенная вариантная замена E3Del в гомозиготном состоянии в гене CYP21A2.

В возрасте 2 лет проведен первый этап феминизирующей пластики (клиторопластика и синусотомия).

В дальнейшем девочка наблюдалась детским эндокринологом, отмечалась удовлетворительная компенсация заболевания. За все время наблюдения кризов надпочечниковой недостаточности не наблюдалось.

Менархе наступило в декретированные сроки (11 лет), в дальнейшем менструальный цикл был регулярным.

При обследовании в возрасте 16 лет конечный рост составлял 160 см (SDS роста 0.42) при целевом среднеродительском росте 162 см, половое развитие соответствовало Таннер 5 (В5, P 4), менструации были регулярными.

В возрасте 16 лет проведен второй этап феминизирующей пластики- интроитопластика кожно-слизистым лоскутом.

Данный клинический пример демонстрирует, что своевременно установленный диагноз по данным неонатального скрининга, даже в случае первоначально неправильно определенной половой принадлежности, позволяет правильно установить пол ребенку, вовремя назначить заместительную гормональную терапию, своевременно провести первый этап феминизирующей пластики. Высокая комплаентность и регулярное наблюдение позволило добиться адекватного течения полового развития и сохранить репродуктивный потенциал у этой пациентки.

## ЗАКЛЮЧЕНИЕ

Проведенный анализ результатов неонатального скрининга ВДКН в Российской Федерации за период 2015–2025 гг. подтверждает высокую эффективность данной программы как ключевого элемента системы охраны здоровья матери и ребенка. За десятилетний период скринингом было охвачено более 15,5 млн новорожденных, что позволило выявить 1871 пациента с ВДКН (дефицитом 21-гидроксилазы). Средняя частота выявления заболевания (1:8309 новорожденных) несколько выше, чем в ряде других стран, и подчеркивает значимость ВДКН как относительно распространенной наследственной патологии, ранняя диагностика которой является жизненно необходимой.

Выявленная существенная вариабельность показателей заболеваемости в различных субъектах и федеральных округах Российской Федерации свидетельствует о гетерогенности генетического ландшафта страны. Особого внимания требуют регионы с высокой заболеваемостью (Карачаево-Черкесская Республика, Ленинградская область, Приморский край), что обосновывает необходимость углубленных популяционно-генетических исследований.

Представленные клинические наблюдения наглядно демонстрируют критическую важность своевременной диагностики. Позднее выявление заболевания ассоциировано с тяжелыми последствиями: необратимым ускорением костного созревания, низкорослостью во взрослом возрасте, формированием тестикулярных образований из эктопированной ткани коры надпочечников (TART) и психосоциальной дезадаптацией, что требует назначения комбинированной и более сложной терапии. В то же время диагностика на основании результатов неонатального скрининга позволяет не только предотвратить развитие жизнеугрожающего сольтеряющего криза, но и обеспечить правильную половую идентификацию, нормальное физическое и половое развитие, а также сохранение репродуктивного потенциала, даже в сложных клинических ситуациях.

Таким образом, клиническая и социальная эффективность неонатального скрининга на ВДКН в Российской Федерации не вызывает сомнений. Дальнейшее совершенствование программы должно быть направлено на минимизацию ложноположительных результатов за счет более широкого внедрения тандемной масс-спектрометрии (ТМС) на втором этапе, унификацию региональных протоколов маршрутизации пациентов, а также на углубление молекулярно-генетических исследований для раннего определения мутации и вместе с ней формы заболевания, а также для оптимизации медико-генетического консультирования семей высокого риска.

## ДОПОЛНИТЕЛЬНАЯ ИНФОРМАЦИЯ

Источники финансирования. Работа выполнена по инициативе авторов без привлечения финансирования.

Конфликт интересов. Авторы декларируют отсутствие явных и потенциальных конфликтов интересов, связанных с содержанием настоящей статьи.
